# Cultural Approach to HIV/AIDS Harm Reduction in Muslim Countries

**DOI:** 10.1186/1477-7517-2-23

**Published:** 2005-10-27

**Authors:** Memoona Hasnain

**Affiliations:** 1 Department of Family Medicine, College of Medicine, University of Illinois at Chicago, Chicago, Illinois, USA

**Keywords:** AIDS, HIV, injection drug users, risk factors, harm reduction, behavioral interventions

## Abstract

Muslim countries, previously considered protected from HIV/AIDS due to religious and cultural norms, are facing a rapidly rising threat. Despite the evidence of an advancing epidemic, the usual response from the policy makers in Muslim countries, for protection against HIV infection, is a major focus on propagating abstention from illicit drug and sexual practices. Sexuality, considered a private matter, is a taboo topic for discussion. Harm reduction, a pragmatic approach for HIV prevention, is underutilized. The social stigma attached to HIV/AIDS, that exists in all societies is much more pronounced in Muslim cultures. This stigma prevents those at risk from coming forward for appropriate counseling, testing, and treatment, as it involves disclosure of risky practices. The purpose of this paper is to define the extent of the HIV/AIDS problem in Muslim countries, outline the major challenges to HIV/AIDS prevention and treatment, and discuss the concept of harm reduction, with a cultural approach, as a strategy to prevent further spread of the disease. Recommendations include integrating HIV prevention and treatment strategies within existing social, cultural and religious frameworks, working with religious leaders as key collaborators, and provision of appropriate healthcare resources and infrastructure for successful HIV prevention and treatment programs in Muslim countries.

## Introduction

AIDS is far more than a medical and biological problem [1]. Around the world, in the year 2003, the AIDS epidemic claimed an estimated three million lives, and almost five million people acquired HIV, 700,000 of them children [2]. The current course of the epidemic is unlikely to change unless the people affected, and those at risk, make a concerted effort to adopt preventive measures. Apart from inadvertent modes of transmission, such as vertical transmission from mother to child and accidental needle stick injuries among health care professionals, certain types of behaviors, such as unprotected sexual intercourse and sharing of hypodermic needles, place individuals at increased risk for HIV and AIDS. The disease is therefore largely avoidable by changes in personal behavior, in other words by voluntary choice. Containment of the AIDS epidemic thus depends on effecting change in behavior and lifestyle to break the chain of transmission. This is all the more challenging because the forces that shape and influence human behavior that is injurious to health are very complex and poorly understood.

In recent years, increasing attention is being paid to the manner in which social and cultural variables influence risk behaviors related to HIV infection transmission. Though the association of contentious ethical and moral issues with HIV risk behaviors exists in all societies, it is much more pronounced in the Muslim world. Thus understanding the role of social and cultural variables affecting HIV transmission in Muslim countries is critical for the development and implementation of successful HIV prevention programs.

*Harm reduction *is a pragmatic philosophy that aims to reduce risks to the individual and the community associated with some often stigmatized, antisocial or illegal behaviors. For HIV/AIDS prevention in Muslim countries, the concept of harm reduction is just as important as in non-Muslim countries. The perspective of harm reduction, developed primarily from work on AIDS and drug problems in the Netherlands [3,4] and United Kingdom [5,6], is a pragmatic approach to the social and individual problems associated with the misuse of psychoactive drugs. In the context of injection drug users, it translates into making sure that if drug misuse cannot be eliminated, some of the problematic risk behaviors leading to HIV transmission, such as sharing of contaminated injection equipment, be reduced. Harm reduction provides a strong rationale for such services as syringe-exchange programs and methadone maintenance treatment.

The ***purpose ***of this paper is to explore the extent of the HIV/AIDS problem in Muslim countries and discuss the modalities of employing a cultural approach as a strategy for harm reduction and, hence, prevention of further spread of the disease.

## The Changing Face of HIV/AIDS: An Emerging Problem in Muslim Countries

The reliability of the available HIV/AIDS incidence, prevalence and mortality data for Muslims is low because many Muslim countries either do not report their statistics or are under-reporting. Global epidemiological indicators, including data from the World Health Organization's Global Health Atlas, do indicate evidence of the burgeoning threat of an HIV/AIDS crisis in Muslim countries. Table 1 provides HIV/AIDS prevalence and AIDS-related mortality data in countries with 50 percent or greater Muslim population, for the period 2001 to 2003. A recent report from the National Bureau of Asian Research in the United States also notes that the ever-growing HIV/AIDS crisis in the Muslim world is a problem that poses potentially serious dangers at the national, regional, and international levels [7].

**Table 1:** HIV/AIDS prevalence and AIDS-related mortality in countries with 50 percent or greater Muslim population, 2001–2003^1^

	Country	Estimated number of adults and children living with HIV/AIDS		Estimated number of deaths due to AIDS	
		**Year**		**Year**	
		**2001**	**2003**	**2001**	**2003**
1	**Afghanistan**	*	*	*	*
2	**Albania**	*	*	*	*
3	**Algeria**	*	<10 000	*	<500
4	**Azerbaijan**	<10 000	<10 000	<500	*
5	**Bahrain**	<10 000	<10 000	*	<500
6	**Bangladesh**	10 000 – <100 000		500 – <1 000	
7	**Brunei Darussalam**		<10 000		<500
8	**Burkina Faso**	100 000 – <500 000	100 000 – <500 000	10 000 – <50 000	10 000 – <50 000
9	**Chad**	100 000 – <500 000	100 000 – <500 000	10 000 – <50 000	10 000 – <50 000
10	**Cocos (Keeling Island)**	*	*	*	*
11	**Comoros**	*	*	*	*
12	**Djibouti**	*	<10 000	*	500 – <1 000
13	**Egypt**	<10 000	10 000 – <100 000	*	500 – <1 000
14	**Eritrea**	10 000 – <100 000	10 000 – <100 000	<500	1 000 – <10 000
15	**Ethiopia**	>=2 M	1 M – <2 M	>=100 000	>=100 000
16	**Gambia**	<10 000	<10 000	<500	500 – <1 000
17	**Gaza Strip**	*	*	*	*
18	**Guinea**	*	100 000 – <500 000	*	1 000 – <10 000
19	**Guinea-Bissau**	10 000 – <100 000	*	1 000 – <10 000	
20	**Indonesia**	100 000 – <500 000	100 000 – <500 000	1 000 – <10 000	1 000 – <10 000
21	**Iran (Islamic Republic of)**	10 000 – <100 000	10 000 – <100 000	<500	500 – <1 000
22	**Iraq**	<10 000	<10 000	*	*
23	**Jordan**	<10 000	<10 000	*	<500
24	**Kazakhstan**	<10 000	10 000 – <100 000	<500	<500
25	**Kuwait**				
26	**Kyrgyzstan**	<10 000	<10 000	<500	<500
27	**Lebanon**		<10 000		<500
28	**Libyan Arab Jamahiriya**	<10 000	10 000 – <100 000	*	*
29	**Malaysia**	10 000 – <100 000	10 000 – <100 000	1 000 – <10 000	1 000 – <10 000
30	**Maldives**	<10 000	*	*	*
31	**Mali**	100 000 – <500 000	100 000 – <500 000	10 000 – <50 000	10 000 – <50 000
32	**Mauritania**	*	<10 000	*	<500
33	**Mayotte**	*	*	*	*
34	**Morocco**	10 000 – <100 000	10 000 – <100 000	*	*
35	**Niger**	*	10 000 – <100 000	*	1 000 – <10 000
36	**Nigeria**	>=2 M	>=2 M	>=100 000	>=100 000
37	**Oman**	<10 000	<10 000	*	<500
38	**Pakistan**	10 000 – <100 000	10 000 – <100 000	1 000 – <10 000	1 000 – <10 000
39	**Qatar**	*	*	*	*
40	**Saudi Arabia**	*	*	*	*
41	**Senegal**	10 000 – <100 000	10 000 – <100 000	1 000 – <10 000	1 000 – <10 000
42	**Sierra Leone**	100 000 – <500 000	*	10 000 – <50 000	*
43	**Somalia**	10 000 – <100 000	*	*	*
44	**Sudan**	100 000 – <500 000	100 000 – <500 000	10 000 – <50 000	10 000 – <50 000
45	**Syrian Arab Republic**	*	<10 000	*	<500
46	**Tajikistan**	<10 000	<10 000	*	<500
47	**Togo**	100 000 – <500 000	100 000 – <500 000	10 000 – <50 000	10 000 – <50 000
48	**Tunisia**	*	<10 000	*	<500
49	**Turkey**	*		*	*
50	**Turkmenistan**	<10 000	<10 000	<500	*
51	**United Arab Emirates**	*	*	*	*
52	**United Republic of Tanzania**	1 M – <2 M	1 M – <2 M	>=100 000	>=100 000
53	**Uzbekistan**	<10 000	10 000 – <100 000	<500	<500
54	**West Bank**	*	*	*	*
55	**Western Sahara**	*	*	*	*
56	**Yemen**	*	*	*	*

^1 ^Sources:

a. For HIV/AIDS statistics: World Health Organization Global Health Atlas 2005, available at:

b. For percentage of Muslim population: CIA World Fact Book 2005, available at:

* Data not available

The continent of Africa, particularly the southern region, continues to have the highest HIV/AIDS incidence and prevalence rates globally [8]. The number of HIV-positive adults range from 6–10% in Nigeria, and 10–18% in Ethiopia; both countries have a majority of residents who are Muslims. By the year 2010, 40% of the African population, where the disease burden is highest, will be Muslim [9]. Some Muslim countries, such as Sudan and Nigeria already show evidence of an explosive epidemic (Table 1). In the Eastern Hemisphere, countries like Kazakhstan, Kyrgyzstan, Tajikistan, Turkmenistan, and Uzbekistan, which were part of the former Soviet Union, face a young and rapidly growing epidemic. East and Southeast Asia, which include countries like China and India containing some of the world's largest populations, show indicators of soon surpassing Africa in terms of their absolute number of cases, if HIV/AIDS rates continue to escalate at their current rate [2]. These projections hold particular relevance for HIV/AIDS in Muslim populations; India and China, though not identified as Muslim countries, have a significant number of Muslims (approximately 138 million Muslims in India, and 40 million in China).

The under-reporting of HIV and AIDS cases in Muslim countries has serious bearings on disease surveillance and monitoring. In the Eastern Mediterranean Region, an estimated 700,000 people are currently living with HIV/AIDS but only 14,198 AIDS cases have been officially registered since the start of the epidemic, indicating under detection, under-reporting, and surveillance difficulties [10]. Of the 22 countries of the region, complete data were lacking for nine countries for 2003, and data from two others had to be discarded because of reporting problems. Although the prevalence of HIV infection among adults in the Eastern Mediterranean Region (0.3%) is roughly equivalent to figures for Western Europe, the number of estimated new HIV/AIDS cases for 2003 is about 60% higher (55,000 in the Eastern Mediterranean Region versus 35,000 in Western Europe), demonstrating the alarming increase in the epidemic in the region [10]. Even though the absolute number of HIV/AIDS cases in the majority of Muslim countries, particularly those in the Middle East or South East Asia, such as Pakistan, may still be lower than other countries; complacency toward this issue will be costly, both in terms of lives and health care costs.

Reasons for the spread of HIV in Muslim countries are open to speculations. Islam places a high value on chaste behavior and prohibits sexual intercourse outside of marriage. It specifically prohibits adultery, homosexuality, and the use of intoxicants [11]. Then how can the spread of HIV/AIDS in Muslim countries be explained? A logical explanation is that in spite of Islamic teachings, some Muslims do engage in activities that lead to acquiring HIV; these risky practices include illicit drug use and/or premarital or extra marital sex. Men who engage in risky behaviors have the potential of transmitting the disease to their unsuspecting wives. Women, on the other hand, also are directly susceptible; in many Muslim countries, brothels and other forms of commercial sex trade are prevalent. The sex workers have poor social support and they are not screened for sexually transmitted diseases including HIV, thus contributing to the spread of infection. Injection drug users (IDUs) also are rapidly becoming a population of increasing concern in the transmission of HIV and AIDS, not only in western countries such as the United States [12-15], but also in developing countries, including Muslim countries. Sex- and drug-related behaviors of IDUs can facilitate HIV transmission even when syringes are not directly shared [15-18].

## Challenges

With regard to curtailing the spread of disease, it is particularly troublesome that a majority of governments in countries with primarily Muslim populations have been slow to respond to the rapidly spreading disease. Despite the evidence of an advancing epidemic, the typical response from the policy makers in Muslim countries is to propagate Muslim ideals, mainly abstention from illicit drug and sexual practices, for protection against HIV infection. Sexuality, considered a private matter, is taboo for discussion. More importantly, there is a denial by most governments in Muslim countries that they are facing an increasing HIV/AIDS threat.

The issue of HIV/AIDS prevention in Muslim countries is a complex problem and requires a multifaceted approach with particular attention to cultural norms. In order to devise harm reduction strategies for HIV prevention in these countries, it is important to study the social dynamics and practices of the populations at risk. Analysis of the cultural context in which risk behaviors occur provides meaningful insight into those factors that shape and define the external reality within which these behaviors take place. Knowledge of why people behave in certain ways and the resources available to them becomes helpful in assisting them to access and utilize available preventive and therapeutic resources. In the context of high-risk groups, it is important to understand that even within them, some individuals choose to indulge in risk behaviors while others do not.

Philipson and Posner [19] note that human actors make rational choices aimed at maximizing the expected utility of the outcome. The subjective welfare of the actor and presence of uncertainty are two inherent components of expected utility maximization. When acquiring information is costly, an uninformed choice – one that underestimates or overestimates the risk to health of some contemplated action – may still be expected utility maximization. Therefore, when education and counseling services are not readily and cheaply available, or when accessing such services means that the user has to disclose risk behaviors and is afraid to do so, he/she has no course but to make uninformed decisions. Effective counseling and education have been shown to change sexual behavior and reduce the risk of HIV transmission even in high-risk groups.

In the context of HIV/AIDS prevention and treatment in Muslim countries, the principles of harm reduction or harm minimization can certainly be utilized to prevent or minimize the spread of HIV infection. However, a clear distinction needs to be made that this approach does not advocate illicit drug and sex related practices. The harm reduction concept which has been successfully applied in substance abusers can also be applied to other high risk groups, such as commercial sex workers. Because consistent condom use has been linked to reductions in HIV seroincidence [15,20,21], and because reductions in frequency of unprotected sex also predict lower levels of HIV infection incidence, the behavioral effects of the intervention carry considerable public health importance. In addition to counseling, IDUs could be provided needles at reduced prices or even free of charge. Regular screenings for sexually transmitted infections (including HIV), and antiretroviral therapy, should make significant contributions to HIV prevention, early detection, and appropriate treatment where required.

In the Muslim World, religion defines culture and the culture gives meaning to every aspect of an individual's life. The following contentious issues need particular attention when designing HIV prevention programs for Muslim countries:

### 1. Gender Inequality

In a majority of Muslim societies, there exists an imbalance in power between men and women, which is apparent in heterosexual relations as well as in the economic and social spheres of life – with men having greater power than women. For most women, the private life within the sanctuary of their houses is their whole life. Women remain uneducated and deprived of resources, making them unaware of their civil, legal and sexual rights, economically vulnerable and largely dependent on men. Due to these inequalities, women are more susceptible to contracting HIV/AIDS as they are less likely to be able to negotiate with their partners infected with HIV/AIDS. Women also are easy targets for abusive relationships and are less able to cope with illness once infected.

### 2. Stigma and Discrimination

The social stigma attached to HIV/AIDS that exists in all societies is much more pronounced in Muslim cultures due to the religious doctrine regarding illicit sex and drug related practices. There are greater negative sanctions for illicit sexual conduct than drug use. Even if there is a suspicion of illicit sexual conduct, the affected person(s) is discriminated against and shunned by the family as well as by the community. The stigma attached to risk behaviors thus prevents those at risk from coming forward for appropriate counseling, testing and treatment, as this would involve disclosure of their risky practices. This results in creating barriers to successful implementation of prevention and treatment strategies where they do exist.

### 3. Ignorance/Misinformation

In developed countries, a majority of the population is aware of the modes of transmission for HIV infection, whereas in the developing countries, misconceptions about the disease and its causes are rampant. Most persons residing in Muslim countries assume that all HIV infections are transmitted only through immoral sexual behaviors and are unaware that it can also be transmitted inadvertently through mother-to-child, accidental pricking of skin and contact with contaminated blood (as in the case of health care professionals) or the possibility of an innocent spouse getting infected by the husband who may have acquired HIV though sexual or drug related contact with other infected persons. Therefore, due to lack of education, expression of compassion towards HIV/AIDS patients is perceived as tolerance towards the practices that lead to acquiring the infection.

### 4. Other issues

In addition to the issues outlined above, the main challenges to instituting an HIV prevention approach include poverty and economic instability, lack of education, wars, internal conflicts, refugees, migrant labor forces, intimidating role of religious leaders and activists, and lack of healthcare resources and infrastructure.

In summary, the existing social, cultural and religious frameworks in Muslim countries do not provide an environment for any safe disclosure for persons who are infected. Hence, the development of effective prevention and support services is often impeded. Meanwhile, growing gender imbalances in HIV rates among women, and the tendency for the virus to be found disproportionately among marginalized and disadvantaged populations throughout the Muslim world, mirror deeply entrenched systems of societal inequality that help to fuel further spread of the epidemic. For those who are not educated, cultural expectations are very difficult to disregard. Containment of the HIV/AIDS epidemic in Muslim countries depends on a combination of individual and community level efforts to effect change in behavior and lifestyle to break the chain of transmission.

## Recommendations

There is an urgent need for developing and implementing policy and programs that provide AIDS education and awareness, prohibit stigmatization, and advocate compassion. Like most religions, Islam condemns homosexuality, drug use, and sex outside of marriage. Though the most important means of protection is obviously abstinence from sex and to remain faithful to the marriage partner, however, Muslims must recognize that in many instances there is a gap between religious teaching and practice; risky behaviors that may not be allowed by Islam are indeed practiced. The main challenge is how to bridge this gap. Religious scholars seem to be divided on the concept of harm reduction. In countries where HIV/AIDS is a rapidly rising threat, such as Uganda [22] and Indonesia [23], religious scholars are taking a more flexible stance and justify the provision and use of condoms and clean needles through Qur'anic and Hadith passages. They reason that the sanctity of life is greater than the sin of condom use and that this strategy can be used as a short term measure,  permissible under a state of emergency. On the other hand, in countries with low incidence and prevalence of HIV/AIDS, religious leaders believe that approving promotion of condoms and clean needles will encourage sexual promiscuity and drug use. To address these controversies, the Organization of Islamic Countries (OIC) should step forward and assume a central role in drafting harm reduction strategies for Muslim countries.

Any effort directed at harm reduction and HIV prevention needs to take into consideration the powerful impact of religious leaders in the community as they play a critical role in Muslim culture. It is important to be cognizant of the reality that religious leaders take issue with harm reduction strategies due to the moral issue involved with the idea of harm reduction. There is a perception  that promoting safe injection and sex related practices will promote illicit drug and sex behaviors. Hence, for HIV prevention programs to be successful, collaboration with religious scholars and leaders is a key element. It is critical to win their confidence and educate them.  Not all cases of HIV and AIDS are contracted through needle exchange or sexual intercourse, and second, regardless of the route of transmission, once a person is infected, he/she should not be treated as a criminal but should be considered a patient suffering from a disease. Just as patients afflicted with any disease deserve the provision of clinical care and support from their family and the society, patients suffering from HIV/AIDS have all the rights to the same services, support and compassion.

Examples of successful prevention efforts that involved religious leaders in Muslim societies include those of Uganda [24] and Senegal [25,26]. In 1992, the Islamic Medical Association of Uganda designed an AIDS prevention project and after conducting a baseline survey prior to community level activities, instituted prevention activities in local Muslim communities. Twenty-three trainers educated over 3,000 religious leaders and their assistants, who in turn educated their communities about AIDS during home visits and at religious gatherings. After two years, there was a significant increase in accurate knowledge of HIV transmission, methods of preventing HIV infection and the risk associated with ablution of the dead and unsterile circumcision. More importantly, there was a significant reduction in self-reported sexual partners among the young respondents of less than 45 years of age. In addition, there was a significant increase in self-reported condom use among males in urban areas [24]. A recent report notes that there is a tangible decline of HIV/AIDS incidence among members of Uganda's Muslim community from 18 percent in the early 90's to the current rate of 6 percent [22].

Senegal also is one of the best examples regarding HIV/AIDS prevention by engaging religious institutions in a proactive role. In March 1990, 260 religious leaders attended a conference on AIDS and reached a consensus to make AIDS control a national priority. Unlike other African countries, HIV/AIDS prevention is a regularly discussed topic in the Friday prayers in mosques in Senegal. From 1989 to 1996, the levels of HIV infection estimated in four sentinel urban regions remained stable at around 1.2 percent in the population of pregnant women, and at three percent in male STD patients [25]. The current 1.2 percent AIDS prevalence rate in the general population of Senegal is in stark contrast to the rest of the continent which has an average AIDS prevalence rate of 30–35 percent. The level of knowledge of preventive practices relating to HIV/AIDS among the general population exceeded 90 percent in the early 1990s.

The reasons for Senegal's successful HIV control are: 1) a good STD tracking system that has been in place since 1969; and 2) AIDS education, utilizing religious institutions and mass media sources such as the radio [26]. From available data, Senegal can rightfully claim to have contained the spread of HIV by intervening early and comprehensively to increase awareness of and knowledge about HIV/AIDS, and to promote safe sexual behaviors via religion and education.

In the context of the AIDS epidemic, limited attention has been paid to the manner in which political, economic and social variables constrain or enable individual behavior related to AIDS [27]. The association of variables such as social capital [28], human capital (educational attainment) [29], and religiosity [30] with HIV risk compels prevention efforts to look beyond the traditional biomedical model of disease prevention. In order to find workable means of combating this disease, research also needs to be directed towards its critically important cultural dimensions [31-33]. The major focus of preventive efforts should be aimed at behavioral change, minimizing the transmission of HIV through unsafe sexual practices and the sharing of contaminated injection drug equipment. The models developed and successfully implemented in western countries can be tailored according to local culture and norms to address the needs of those at risk of or suffering from HIV/AIDS in Muslim countries. In addition to proper food, housing, education, employment, regardless of country of residence, all persons at risk of or suffering from HIV/AIDS should have the right to safe disclosure and appropriate health care.

There also is an urgent need in Muslim countries for increasing infection surveillance and enhancing HIV preventive and therapeutic services for high-risk groups, such as commercial sex workers, drug abusers, and those with alternative sexual lifestyles, not simply those who identify themselves as being either infected or possibly infected. In addition, legislative and social changes, such as protecting the legal rights of the infected, promoting safer alternative behaviors among high-risk groups, and spreading the message that being a good Muslim can include taking care of those infected by HIV would be helpful in combating the spread of the disease. HIV/AIDS education and control efforts could also become part of each citizen's duty. The international community can also assist by helping poorer countries establish social programs or simply sharing experience in drug treatment and behavioral change efforts [7].

In ***summary***, our recommendations to stem the spread of HIV in Muslim countries include:

1. Addressing the underlying societal problems such as poverty, lack of education and gender imbalance;

2. Developing collaborative prevention and care models (including all possible stakeholders such as, religious scholars, academics, expert health professionals, policy makers, non governmental organization, community based organizations, and HIV positive persons);

3. Development and provision of appropriate healthcare resources and infrastructure including:

• Blood safety and infection control

• Appropriate surveillance and reporting mechanisms

• Drug abuse prevention and rehabilitation services

• Medical care and social support including HIV counseling, testing and treatment facilities

• Adequate number of trained health care workforce

• Appropriate reproductive health care programs

• Broader efforts directed at enhancing information, education and communication.

## Conclusion

The challenge of addressing the rising threat of the spread of HIV/AIDS in Muslim countries/societies is significant. The most effective public health method of controlling the spread of AIDS is education and changing the way people behave. Political, financial, and social barriers have often kept the most effective prevention and treatment strategies from reaching persons at the highest risk. There is a need to ensure sustained access to preventive and treatment services for all high-risk groups. The goal of prevention is best achieved through an ongoing process, open to change and flexible to adaptation. Incorporating such change within religious and cultural frameworks is no easy task. This is the challenge we are facing and it is up to us, individually and collectively, as health care professionals and researchers to respond.

To ensure ongoing usefulness of public health policies related to HIV prevention, we must learn to synthesize old knowledge with new, and, at the same time, utilize opportunities to choose new directions. The framework proposed in this paper can serve as an initial model for appropriate HIV prevention and care programs in Muslim countries. Risk needs to be viewed within the context of the social subculture of Muslim countries to design strategies to reduce risk behaviors related to HIV transmission. The social dimension of health mandates that policy and program measures to stop AIDS be a balance of social and biomedical scientific efforts. Our recommendations include education, involvement and mobilization of diverse stakeholders, particularly religious leaders; establishing sustainable financing for AIDS treatment and drug procurement; institutingregulatory mechanisms to ensure blood safety and appropriate delivery of HIV/AIDS counseling, screening and treatment services; improvement in health infrastructure; and training of health care workers. None of the above will be successful without reducing the stigma associated with HIV and AIDS, developing compassion for those afflicted, and designing harm reduction strategies which would be conceptually integrated within the existing social, cultural, and religious frameworks in Muslim countries.
